# Assessing the Quality of Orthopaedic Operation Notes in Accordance With the Royal College of Surgeons Guidelines: An Audit Cycle

**DOI:** 10.7759/cureus.9707

**Published:** 2020-08-12

**Authors:** Mohamed Kamal Elbashir Mustafa, Alaa Mohamed Mohamed Khairy, Ahmed Bashir Elmadani Ahmed

**Affiliations:** 1 Vascular Surgery, University Hospital Galway, Galway, IRL; 2 Orthopaedic Surgery, Ribat University Hospital, Khartoum, SDN

**Keywords:** orthopeadics, audit, operative notes

## Abstract

Introduction

Accurate and detailed operation notes are of great importance in all surgical specialties not only for patient care but also for providing information for research, audit and medico-legal purposes. In this audit cycle, we assessed the quality of operation notes against the standards set by the Royal College of Surgeons of England.

Methodology

A sample of 59 operation notes was randomly selected from the orthopaedics department at Ribat University Hospital and retrospectively audited by three reviewers according to the Royal College of Surgeons of England Good Surgical Practice guidelines released in 2014. A memory aid was then placed in the operation theatre, emphasising mainly the points with poor compliance in the audit. A re-audit was then performed for another 59 operation notes.

Results

During the first audit, 59 elective operation notes were reviewed, and there was good compliance with date documentation (86%), diagnosis (85%), operating surgeon (90%), assistants’ names (86%), operative procedure (98%), detailed post-operative instructions (98%) and the signature (75%). In the re-audit phase, another 59 operative notes were reviewed; four of them were emergency operations. An improvement was noted in documenting the information that had been poorly documented in the first audit. In the first audit, 20% of the operation notes were written by the operating surgeon, while only 14% were written by the operating surgeon in the re-audit.

Conclusion

Our implementation of a memory aid in the operation theatre helped to improve the reporting of some of the criteria; however, some components of the operation notes remained poorly filled in.

## Introduction

Among the many responsibilities of a physician, the first and foremost is to his patients and their health and safety. A very important part of good medical practice is to maintain patient records. The General Medical Council recommends ensuring accurate, comprehensive and legible records are maintained for every patient by the surgeon [1]. Accurate and detailed operation notes are of great importance in all surgical specialties not only for patient care but also for providing information for research, audit and medico-legal purposes [2].

Operative notes are often presented in legal malpractice cases, and studies have demonstrated that up to 45% of operative notes are indefensible from a medico-legal standpoint. Incomplete and illegible notes are often a source of weakness in the defence of surgeons in courts [3]. Clear and legible notes relating to all surgical procedures are therefore extremely important [4]. Handwritten notes are still used worldwide; however, establishing their legibility could often be a major drawback [5]. An audit conducted in Kuwait by Sweed et al. demonstrated that 20% of the orthopaedics operative notes they reviewed contained illegible parts, was incomplete and included confusing abbreviations [6].

In order to improve our clinical practice, there is a need to adopt a standardised way to document operative notes, so that our records contain all the details necessary to give patients the best possible care. Although there are no known standardised guidelines in place in Sudan relating to operative notes, there are international guidelines that are in use and are well recognised, such as those set by the Royal College of Surgeons of England [7]. Therefore, in this study, we assess the quality of operative notes against the standards set by Royal College of Surgeons of England, with a view to improve the quality of operative notes and ensure quality patient care. We also aim to test the applicability of these guidelines in the setting of a developing country with a high workload and limited medical technology.

## Materials and methods

Our institute is a tertiary care hospital based in Khartoum, the capital of Sudan. A sample of 59 operation notes collected between March 1, 2019, to April 1, 2019, were randomly selected from the orthopaedic department at the Ribat University Hospital in Khartoum state, Sudan; they were retrospectively audited by three reviewers according to the Royal College of Surgeons of England Good Surgical Practice guidelines (Table 1). The Operative notes were all based on the standard template used at the Ribat University Hospital.

**Table 1:** Operation notes standard criteria as set by the Royal College of Surgeons of England

Serial no.	Details
1	Date and time
2	Elective/emergency procedure
3	The names of the operating surgeon and assistant
4	The operative procedure carried out
5	The incision
6	The operative diagnosis
7	The operative findings
8	Any problems/complications
9	Any extra procedure performed and the reason why it was performed
10	Details of tissue removed, added or altered
11	Identification of any prosthesis used, including the serial numbers of prostheses and other implanted materials
12	Details of closure technique
13	Post-operative care instructions
14	A signature

The notes were audited in accordance with the Royal College of Surgeons of England Good Surgical Practice guidelines [7], with additional items related to orthopaedic surgery making up 19 items in total: the diagnosis, elective/emergency, date and time of surgery, operating surgeon, assistants’ names, anaesthetist's name, operative procedure, incision, operative diagnosis, operative findings, problems/complications, details of prosthesis/material used (including serial number), details of closure technique, anticipated blood loss, drain, tourniquet time, detailed post-op instructions, and the signature. Also, we audited who wrote the operation note, and whether it was written by the operating surgeon or not.

**Figure 1:**
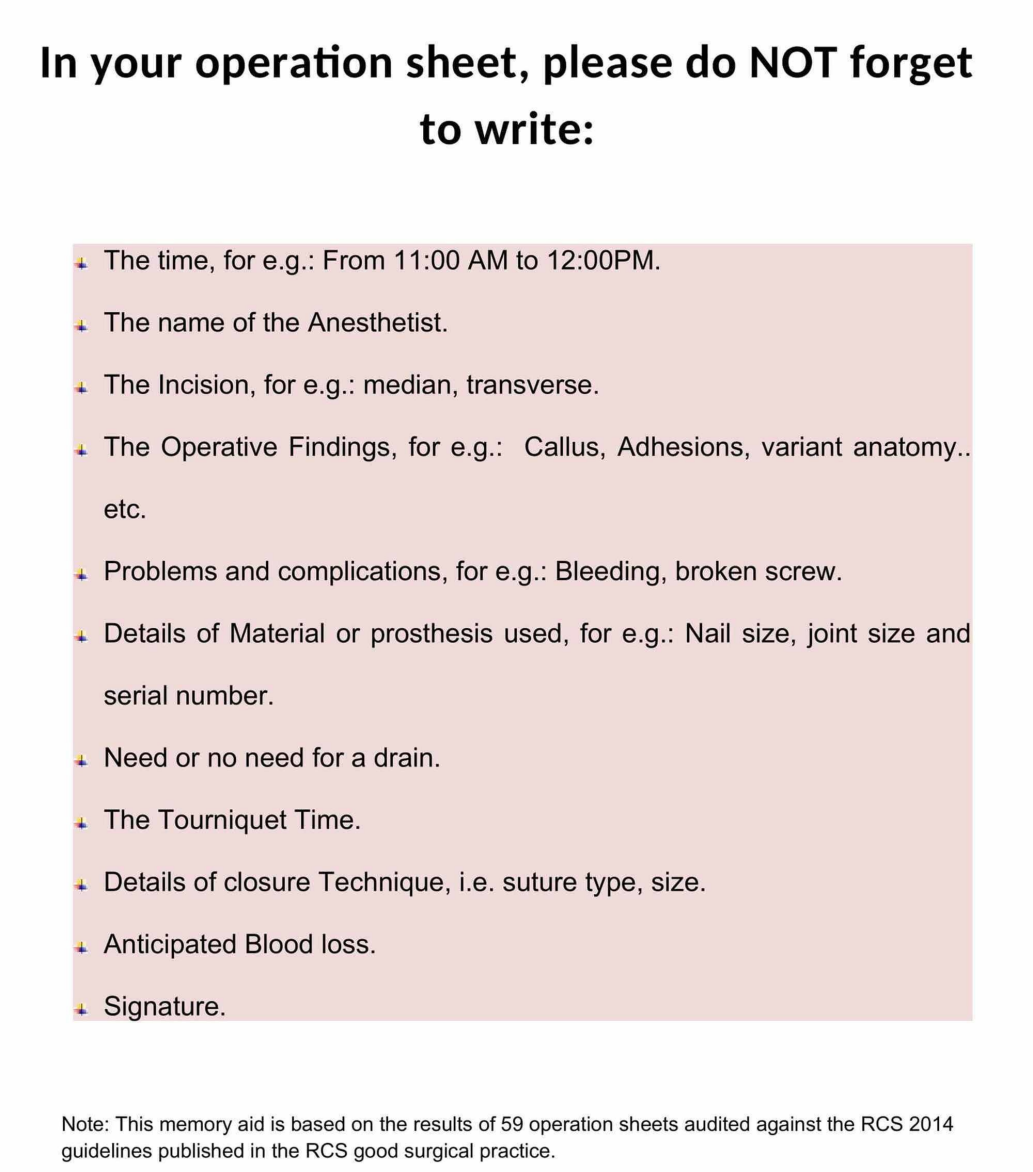
Memory aid used during the re-audit phase RCS: Royal College of Surgeons

After the descriptive analysis of the audit data using Microsoft Excel version 2010 (Microsoft Corporation, Redmond, WA), a memory aid was then placed in the operation theatre during May-June 2019, emphasising mainly the points with poor compliance (Figure 1). A re-audit was then performed for another 59 operation notes, with regard to the same items.

## Results

During the first half of the audit cycle, 59 operation notes of surgeries performed between March 1, 2019, to April 1, 2019, were retrospectively reviewed against the guidelines for the operation notes set by the Royal College of Surgeons of England in the recent 2014 edition; there was good compliance with respect to date documentation (86%), diagnosis (85%), name of operating surgeon (90%), assistants’ names (86%), description of the operative procedure (98%), detailed post-operative instructions (98%) and the signature (75%). The audit highlighted five components with poor compliance: the time or length of the operation, the name of the anaesthetist, the incision type or shape, usage of drains and details of the prosthesis used such as the serial number or size. A memory aid was then designed and placed in the operation room for two months, emphasising on the poorly documented aspects (Figure 1).

**Figure 2:**
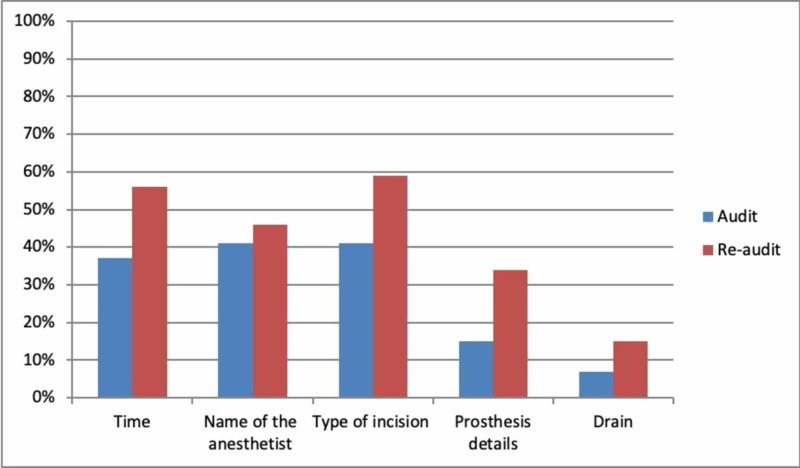
Improvements in operation sheet elements after the use of memory aid

In the re-audit phase, another 59 operative notes were reviewed of which four were emergency operations. An improvement was noted in documenting the information that had been poorly documented in the first audit. Time documentation improved from 37% to 56% of the audited notes, documenting the name of the anaesthetist improved slightly to 46% from 41%, and documenting the type of incision rose to 59% from 41%. In the operations where a prosthesis was used, the size and details of the prosthesis reported improved by more than half, from 15% to 34%. Likewise, reporting on the insertion of the drain in its right place on the operation sheet improved to 15% from 7% (Figure 2).

In the first audit, 20% of the operation notes were recorded by the operating surgeon, while only 14% were documented by the operating surgeon in the re-audit phase.

## Discussion

Meticulous record-keeping with regard to patient notes is becoming more important in medical practice, especially in a setting where complaints relating to medical negligence and malpractice cases are on the rise; but first and foremost, operative notes are an essential pillar in patient care as well as education and research. This audit examined one of the most important documents in medical practice in general and surgical care in particular.

In our hospital, there is a single, standard operation sheet that is shared among all surgical specialties. In the audit we conducted, it seemed feasible to use a memory aid as a method to improve the quality of documentation in the operation notes. Din et al. used a similar approach in their audit of orthopaedic operation notes, which included elective operations as well as emergency surgeries; their use of memory aid improved the overall documentation of details from 90% to 97.1% of the notes [8]. These results and the results from our audit prove that implementing a simple measure such as placing a memory aid in the operation theatre can affect patient care significantly in a positive way. Shayah et al. also used memory aids in the ear, nose and throat (ENT) theatres, and in their re-audit cycle, all the components of operation notes improved by 100% [9].

On the other hand, Fionn et al. suggested the use of typed, orthopaedic-specific operation notes and tested them in their audit in comparison to handwritten notes [10]. Their audit showed that typed notes had better legibility compared to the handwritten ones, and resulted in better documentation as they had added checks related to orthopaedic surgeries like tourniquet time and prosthesis details. Similarly, Andrew et al. used electronic, custom-made operation notes [11]. However, they were procedure-specific, specifically related to hip arthroplasty. They demonstrated that the electronic template raised the general accuracy of operation notes from 58% to 92%, improved documentation as per all the specific Royal College of Surgeons parameters and reduced variations seen with handwritten notes. Nevertheless, the applicability of computerised or procedure-specific operation notes is very limited in many settings, even in developed countries, since many hospitals do not have a fully computerised system with regard to patient medical records. Ghani et al. used a smarter electronic operation note for orthopaedic surgeries in general, and this modern intervention demonstrated 100% accuracy and readability of the notes.

Many other audits and studies have used similar methods like pre-typed form or checklists. All methods aim to improve the quality and documentation of operation notes in different surgical specialties, bearing in mind the importance of these records in patient care. In our view, the best method to improve the quality of operation notes is the use of electronic forms that take into account the differences between various surgical specialties; however, the applicability of this method in Sudan is difficult at present; nevertheless, it should be considered as a plan to be implemented in the near future as medical practice in general is becoming more digitalised. For the time being, we highly recommend modifying the standard operation notes to be tailored according to the surgical specialty in question; for example, adding headings related to tourniquet time and prostheses details in orthopaedic patient files will significantly improve the adherence by surgeons to these parameters.

## Conclusions

The proforma used in our hospital aligns well with the criteria described in the Royal College of Surgeons Good Surgical Practice guidelines released in 2014. Our implementation of a memory aid in the operation theatre helped to improve the reporting of some of the criteria; however, some components of the operation notes remained poorly filled in. We strongly recommend continuing with the implementation of audits in our clinical practice among trainees and departments in order to closely analyse our practices, especially with regard to patient safety and quality of care.
